# Beyond the left-right brain divide: a framework for dual-domain cognitive fluency

**DOI:** 10.3389/fcogn.2026.1727422

**Published:** 2026-05-07

**Authors:** Don A. Affognon

**Affiliations:** Department of Educational Psychology, University of Nevada, Las Vegas, NV, United States

**Keywords:** cognitive flexibility, dual-domain fluency, executive function, representational fluency, symbolic reasoning

## Abstract

Longstanding distinctions between “verbal” and “mathematical” minds continue to shape educational assessment, curriculum design, and how learners are categorized by perceived cognitive strengths. Yet plenty of evidence from psychology and cognitive neuroscience points to a more unified model of intelligence that refutes the false dichotomy. In this article, I propose dual-domain cognitive fluency (DDCF) as a way to describe how the human mind can move fluidly between words and numbers and derive meaning from both. Building on research in symbolic cognition, executive function, and cognitive flexibility, this article identifies three dimensions: symbolic translation between modalities, cognitive flexibility across task demands, and layered reasoning, which integrates propositional logic with linguistic abstraction. DDCF captures the symbolic agility now required in knowledge-based environments where the integration of representational systems matters more than isolated domain expertise. Examples range from data storytelling in journalism to algebraic modeling in the social sciences—contexts in which verbal and quantitative reasoning operate in tandem. These insights matter for pedagogy, curriculum design, and the evolving demands of data-intensive workplaces. Far from a niche ability, dual-domain fluency reflects a generalizable cognitive capacity that existing models of intelligence have struggled to measure, reward, or systematically support.

## 1 Introduction

The myth of “left-brained” and “right-brained” thinkers persists in schools, workplaces, and cultural narratives, although neuroscience has consistently discredited it. Students are still sorted into verbal or numerical tracks (Wang and Degol, 2017). Standardized assessments reward domain-specific strength over integrative ability (Rose et al., 2013). This dichotomy also appears in professional discourse, where verbal communication and quantitative fluency are often treated as separate strengths rather than coordinated capacities. But neuroimaging research (Nielsen et al., 2013), hemispheric specialization (Toga and Thompson, 2003), and cognitive psychology research (Prat et al., 2016) have shown that real-world intelligence defies such rigid partitions. Despite these widespread assumptions, the brain does not compartmentalize language and math; it connects them in ways that support fluid reasoning across both. In this article, I argue that dual-domain cognitive fluency (DDCF) offers a framework for understanding intelligence that bridges the outdated gap between verbal and mathematical ability. DDCF reflects the ability to interpret symbols, shift mental schemas, and reason with numbers and words.

Although educators and cognitive scientists have long recognized that learners exhibit diverse cognitive profiles (Gardner, 2006), the institutional design of modern schooling still defaults to binary categorization. Students are tracked, tested, and instructed as if cognitive specialization precludes representational integration (de Ree et al., 2023). Academic interventions target “math learners” or “reading learners” as if the two could not be mutually reinforced (Sievertsen, 2022). As a consequence, systems intended to cultivate cognitive potential constrain the development of symbolic flexibility instead. When schools fail to promote integrative reasoning, they run the explicit risk of misidentifying mental agility as inconsistency, and cognitive range as fragmentation. In music, for example, range is celebrated, not penalized. Pedagogical research emphasizes that smooth register transitions—moving fluidly between chest and head voice—are recognized as signs of mastery, not inconsistency or fragmentation. And practices such as those developed by Roy Hart openly train performers to expand vocal range as a form of expressive mastery (Crawford and Pikes, 2019). Should the same recognition not apply to learners who shift fluently across symbolic systems?

Dual-domain cognitive fluency encapsulates a deep-seated aptitude for symbolic reasoning shaped by millennia of intellectual practice. The ability to transition between quantitative structure and semantic meaning has driven insights in academic disciplines ranging from economics to epidemiology (Ralph et al., 2017). Today, in a world saturated with data and marked by disciplinary convergence, integrative thinking has become indispensable because it enables people to extract meaning from statistics and impose clarity on complexity. As artificial intelligence accelerates the automation of specialized tasks, the cognitive advantage now belongs to those who can fluidly translate across representational systems (Schunn and Silk, 2011).

Whereas verbal and mathematical abilities have been studied extensively as discrete constructs (Carroll, 1993; Deary et al., 2010), their coordinated deployment remains undertheorized, even though this hybrid aptitude is increasingly essential in fields as varied as interdisciplinary research, data journalism, and policy analysis. Indeed, fluency across modalities is no longer confined to polymaths. It has become a prerequisite for advanced reasoning in knowledge-based economies. It follows that the purpose of this paper has three objectives:

(1) To define dual-domain cognitive fluency as a unique cognitive framework.
(2) To situate it within existing models of symbolic reasoning and executive control (Friedman and Miyake, 2017).
(3) To consider its implications for education and work.

By challenging the dichotomy between verbal and numerical ability, this article advances an integrative view of cognition not as a deviation but as an inherent and measurable human aptitude.

## 2 Rethinking cognitive fluency: theory and evidence

### 2.1 Theoretical dimensions

The human mind operates as a multiplex of evolved systems, some ancient and automatic, others recent and deliberative, each designed to handle specific domains such as objects, agents, numbers, and language (Carey, 2009; Spelke and Kinzler, 2007). One of these systems supports movement between words and numbers is the aptitude to shuttle between words and numbers, narratives and notations, concepts and calculations. This cognitive ability, introduced earlier as dual-domain cognitive fluency (DDCF), challenges the widespread classification of people as either “verbal” or “quantitative” and captures the human aptitude for symbolic transformation across representational systems. Rooted in cognitive flexibility and compositional abstraction, DDCF is not just a skill but a deep structure of reasoning that enables the synthesis of semantically rich language with the concision of symbolic logic (Barbey et al., 2013; Monti et al., 2009). In cognitive science, this aptitude falls under the umbrella of symbolic reasoning, a cognitive function involved in numerical and linguistic inference (Goel, 2007; Monti et al., 2009). Although DDCF represents a core structure of higher reasoning, it remains largely absent from how schools and workplaces evaluate or reward intellectual ability.

### 2.2 Dual-domain fluency in context

Even though dual-domain fluency reflects a foundational way in which the human mind organizes and manipulates information, it remains largely underrepresented in major theoretical models. From Spearman's concept of a general intelligence factor (*g*) to Carroll's three-stratum model, historical theories of intelligence have consistently privileged monolithic constructs over integrative abilities (Carroll, 1993; Jensen, 1998; Spearman, 1904). Nevertheless, real-world problem solving often draws less from unidimensional aptitude and more from the ability to recode, reframe, and recontextualize symbolic information across domains. Neuroimaging studies suggest that such cross-modal reasoning activates distinct but interconnected brain regions—in particular, the left inferior frontal gyrus for semantic encoding, the bilateral intraparietal sulcus for numerical abstraction, and the dorsolateral prefrontal cortex for cognitive control (Fedorenko et al., 2013; Supekar et al., 2013). These findings support models of intelligence that emphasize integrative representational fluency over isolated domain strength. Perhaps more important is the fact that cross-domain reasoning is not just an artifact of high IQ. It represents a qualitatively different cognitive function that maintains all linguistic, quantitative, and executive systems in dynamic coordination (Barbey et al., 2013; Chuderski et al., 2012). This defines the structural core of dual-domain cognitive fluency: transduction across partitions. The neglect of integrative cognition finds sharp expression in labor markets, where educational paradigms lag behind the hybrid symbolic demands of knowledge-intensive jobs (see Table 1).

**Table 1:** Symbolic integration demand and institutional recognition across domains.

Symbolic integration demand	Low institutional recognition	High institutional recognition
**High symbolic integration demand**	**Core mismatch:** tasks require coordinated verbal + quantitative reasoning but are not recognized as such (e.g., policy analysis, journalism, behavioral economics).	Rare cases where integrative reasoning is both required and recognized.
**Low symbolic integration demand**	Baseline tasks with limited symbolic coordination.	Tasks framed as technical or quantitative and strongly credentialed.

The table shows a systematic asymmetry between symbolic integration demands and institutional recognition. The most consequential region lies in domains that require sustained coordination across numerical, verbal, and visual systems yet remain weakly identified within prevailing evaluative frameworks. These domains demand continuous translation across symbolic forms, but are often classified as primarily verbal or qualitative, obscuring the cognitive operations they require. By contrast, domains organized around single-system proficiency are more readily aligned with institutional categories and receive clearer forms of validation. This pattern reveals that dual-domain fluency is not marginal but structurally present in high-demand cognitive environments, even as it remains under-recognized within conventional systems of credentialing and assessment.

A key aspect of cross-domain thinking is symbolic translation: the skill of shifting an idea from numbers into language, or vice versa, without losing its meaning. This capacity underlies everything from science communication to mathematical modeling and policy design. Research on representational competence indicates that people trained to interpret and produce multiple symbol systems outperform peers in both STEM and humanities contexts (Ainsworth, 2006; Geary et al., 2017). Neurocognitive studies also indicate that symbolic translation draws upon joint activation of classical language areas, such as Broca's and Wernicke's areas, and numerical-processing centers such as the intraparietal sulcus—indicating a multi-system convergence rather than an isolated function (De Smedt et al., 2011; Prado et al., 2011). However, this process cannot be reduced solely to working memory or verbal intelligence. It entails the alignment of structurally distinct representations through relational mapping and analogical abstraction—core features of analogical reasoning that enable learners to move fluidly between symbolic codes and conceptual schemas (Gentner and Smith, 2012; Richland et al., 2007). This ability to align structurally distinct representations enables conceptual clarity across domains. Without it, interdisciplinary reasoning collapses into domain-bound terminology inaccessible to any outsiders (Gentner and Forbus, 2011; Weinberger et al., 2022).

Symbolic translation presupposes cognitive flexibility, or the ability to toggle among mental sets, perceptual frames, and reasoning strategies as task demands evolve. This is not a character trait, but a well-designed executive function underpinned by identifiable neural substrates and shaped by a distinct maturational sequence (Diamond, 2013; Zelazo et al., 2003). Research in both clinical and normative populations shows that cognitive flexibility predicts transfer learning, effective problem-solving in unfamiliar situations, and the generalization of abstract rules across domains (Ionescu, 2012). Unlike attentional shifting, cognitive flexibility involves rule reassignment and modality switching, not merely the redirection of focus (Andrews-Hanna et al., 2014). MRI research also links cognitive flexibility to coordinated activity between the anterior cingulate cortex and lateral prefrontal regions, neural circuits implicated in conflict monitoring, goal updating, and cognitive reconfiguration. This coordination facilitates the cognitive flexibility that drives dual-domain fluency in symbolic tasks (Liston et al., 2006). Given the human mind's disposition for symbolic translation and cognitive flexibility, the third component of DDCF, layered reasoning, involves the recursive integration of formal structure with contextual meaning, enabling individuals to reconcile abstract logic and interpretive depth. It is the process by which formal logic is reconciled with context-rich semantics—for example, to enable the learner to assess the validity of an argument as well as its relevance and meaning. According to (Goel et al. 2004) and (Reverberi et al. 2007), this kind of reasoning activates the medial prefrontal cortex (for abstract rule application) and the posterior cingulate (for semantic self-relevance assessment), demonstrating a deep interplay between logic and meaning. Most important, layered reasoning underlies argument evaluation in disciplines such as law, ethics, and mathematics, where symbols cannot be separated from interpretation (Carpenter et al., 1990; Stenning and Van Lambalgen, 2008). Experimental studies in educational psychology further show that when students are trained to map logical structures onto semantic content, through argument diagrams and symbolic scaffolds, their reasoning gets sharper and more transferable (Feeney et al., 2004; see also Kuhn and Udell, 2003).

A classic example from mathematics illustrates how the three components of dual-domain fluency can converge in practice. Consider the Pythagorean theorem. A student who understands it fluently can translate between its algebraic form, (*a*^2^ + *b*^2^ = *c*^2^), its geometric representation (right-angled triangles), and the verbal reasoning behind it (“the square of the hypotenuse equals the sum of the squares of the other two sides”). But to apply the theorem to unfamiliar contexts, such as estimating diagonal distances in architecture or mapping software, requires symbolic translation as well as cognitive flexibility and layered reasoning. To arrive at the solution, the student must recognize the structural form and understand its relevance within the new context (Carpenter et al., 1990; Kuhn and Udell, 2003; see also Van Gelder et al., 2004). Before turning to the empirical evidence supporting this framework, it matters to distinguish dual-domain cognitive fluency from another framework with which it might be confused.

Grounded in associative and imagery-based learning models, dual coding theory (DCT) emphasizes the encoding of information in two sensory modalities (verbal and visual) to support retention and comprehension (Clark and Paivio, 1991; Paivio, 1990). Unlike DDCF, which emphasizes the learner's ability to translate between symbolic systems and maintain structural coherence while doing so, the central premise of DCT is that when learners are exposed to both text and image, they generate dual memory traces that enhance recall through complementary channels. Whereas DCT seeks to strengthen associative memory, DDCF prioritizes the capacity for abstract mapping across representational formats. DCT presumes that integration occurs when verbal and pictorial representations are co-presented and mutually reinforcing, typically at the perceptual or mnemonic level. By comparison, DDCF entails alignment of structurally distinct but conceptually equivalent symbolic forms, as when a learner translates a verbal statement into an algebraic equation or maps a logical claim onto a visual model. This process draws on executive function, symbolic abstraction, and analogical reasoning (Gentner, 1983; Miyake and Friedman, 2012; Monti et al., 2009). Unlike DCT, which enhances learning through dual encoding, DDCF supports reasoning by enabling structural transformation across modalities. The difference between these two frameworks is clear: DCT enriches representational input, while DDCF transforms it. In the context of DDCF, transformation refers to a structure-preserving recoding operation in which an idea expressed in one symbolic format is systematically re-expressed in another while maintaining relational invariants. For example, a causal narrative about economic inflation can be translated into a mathematical model, then rearticulated as a policy explanation without loss of the underlying relational structure. Such transformations require learners to identify the conceptual relationships embedded in a representation and reconstruct them across alternative symbolic systems, a process closely aligned with structural mapping theory and analogical reasoning (Gentner and Smith, 2012; Holyoak, 2012). Rather than merely strengthening memory traces through dual encoding, as proposed by dual coding theory, DDCF involves the active reorganization of symbolic representations in ways that preserve logical structure while altering representational form. Finally, while DCT builds primarily upon associative learning and dual-channel memory encoding principles rooted in cognitive psychology, DDCF emerges from a different lineage altogether. It builds upon executive function research, structural mapping theory, and symbolic reasoning literature, all of which emphasize dynamic coordination, rule-governed abstraction, and mental flexibility.

### 2.3 Fluency across domains

The foregoing example embeds a greater point: the three dimensions of dual-domain fluency (symbolic translation, cognitive flexibility, and layered reasoning) do not function in isolation but in synchrony. Empirical studies of problem-solving in STEM show that experts constantly shift between verbal reasoning, visual models, and symbolic manipulations, often within seconds (Ainsworth et al., 2011; Stieff et al., 2005). This fluidity is not merely a byproduct of expertise; it is a defining characteristic. Neurocognitive research confirms that expert reasoning involves rapid shifts across verbal, visual, and symbolic modalities that activate neural hubs responsible for relational integration. Chief among these is the rostrolateral prefrontal cortex, which supports abstract, multi-representational thought (Christoff et al., 2001; Dumontheil, 2014). Educational research further indicates that when students are explicitly taught to coordinate representations, such as translating word problems into diagrams or graphs, their ability to transfer knowledge improves, as does their metacognitive awareness (Koedinger and Nathan, 2004; Seufert, 2003). Likewise, recent syntheses confirm that instructional designs incorporating multiple external representations significantly enhance conceptual understanding, representational competence, and knowledge transfer across domains (Ainsworth, 2006; Rau, 2017; Seufert, 2019). This convergence of neural and pedagogical evidence confirms that DDCF is not merely another conceptual model. Contemporary syntheses of learning with multiple representations similarly show that coordinated representational use strengthens conceptual integration and supports transfer across disciplinary contexts, reinforcing the claim that symbolic coordination constitutes a core mechanism of higher-order reasoning (Rau, 2017; Seufert, 2019). It constitutes a teachable set of cognitive strategies grounded in both brain function and pedagogic practice. Such converging evidence anchors DDCF in empirical and instructional realities, positions it for theoretical scrutiny, and raises the question whether it constitutes a genuinely new framework or reformulates existing models of intelligence. Critics may argue that DDCF is a rebranding of intelligence, or an abstraction that is too vague for critical examination. But such criticism would conflate taxonomic overlap with conceptual redundancy. The reason is that traditional measures of general intelligence tend to assess modular processing (verbal analogies in one section, matrix reasoning in another, and spatial rotation in a third). In contrast, DDCF captures what such measures overlook: the ability to reconfigure ideas across representational systems and decide when cross-domain translation clarifies rather than distorts (Kovacs and Conway, 2016; McGill and Dombrowski, 2019). Moreover, several emerging models of cognition, such as process overlap theory and mutualism, support the notion that integrative abilities may explain more real-world differences than domain-specific strengths (Kievit et al., 2017; see also Van Der Maas et al., 2006). Viewed through this lens, DDCF is not a new label for an old framework but rather a more inclusive definition of cognitive competence. In this context, inclusiveness refers to the integration of symbolic operations that traditionally fall under separate ability constructs. Rather than measuring verbal reasoning, quantitative reasoning, or executive control in isolation, DDCF conceptualizes cognitive competence as the coordinated capacity to translate, align, and reinterpret symbolic representations across domains. This integrative perspective therefore expands the scope of cognitive competence beyond domain-specific performance and emphasizes the ability to maintain relational structure while navigating between symbolic systems (Kovacs and Conway, 2016; Kievit et al., 2017).

The long-standing division between “verbal” and “quantitative” thinkers has shaped educational practice and research, even though neuroscientific evidence shows that complex reasoning draws on shared, bilateral networks across domains (Jung and Haier, 2007). Several functional connectivity studies using resting-state and task-based MRI consistently suggest that complex reasoning activates bilateral networks—especially within the frontoparietal and cingulo-opercular systems—irrespective of domain label (Cole et al., 2014; Jung and Haier, 2007). Even tasks clearly designed to be “mathematical” or “linguistic” frequently draw on overlapping cortical pathways, when symbolic complexity is high (Amalric and Dehaene, 2018; Fedorenko and Thompson-Schill, 2014). Supplementary findings from educational research affirm that students who demonstrate integrative fluency across verbal and numerical modalities outperform their peers on far-transfer evaluations and interdisciplinary problem-solving tasks (Rittle-Johnson and Schneider, 2014; Zamarian et al., 2009). What DDCF provides, then, is not a bridge across representational modes, but a means for capturing how integrated reasoning enables performance across domains.

Viewed in aggregate, DDCF represents a form of higher-order cognition characterized by the ability to translate across symbolic systems, adapt across modalities, and reason abstractly while integrating contextual information. Studies in workplace cognition have shown that top performers in data-intensive professions are often those who combine analytical rigor with communicative precision—those who can explain a complex model in a sentence and explain a sentence with a model (Autor et al., 2003; National Research Council, 2012). In school settings, students who acquire representational fluency outperform their peers on standardized tests in their ability to apply knowledge to new situations (Bransford et al., 2000; Collins and Kapur, 2014). Given that human cognition evolved to integrate different forms of meaning, educational and professional institutions should cultivate and reward this integrative ability (Donald, 1993; Gabora and Smith, 2018).

### 2.4 The role of executive function

The cornerstone of dual-domain cognitive fluency is not domain-specific aptitude but executive function—those higher-order processes that allow people to monitor conflict, switch mental sets, and update goals in response to changing demands. In their landmark latent-variable analysis, (Miyake and Friedman 2012) found that the components of executive function (shifting between tasks, updating working memory representations, and inhibiting prepotent responses) operate independently and in tandem. Within the DDCF framework, executive function operates as the control architecture that coordinates symbolic transformations across representational systems. Cognitive shifting enables transitions between symbolic formats (e.g., verbal, mathematical, or graphical), working-memory updating maintains relational invariants during representational recoding, and inhibitory control suppresses misleading surface features that could distort structural alignment. Together, these executive processes stabilize symbolic translation and layered reasoning, ensuring that conceptual relationships remain intact while representational form changes (Diamond, 2013; Friedman and Robbins, 2022).

However, this pattern of interdependence does not reflect compartmentalized skills. Instead, it reflects a functional architecture that allows the mind to reconfigure symbolic representations across domains without losing structural coherence. The cognitive skill required to translate a verbal argument into an algebraic equality, or to use narrative explanation to reinterpret a statistical model, depends on such a flexible orchestration. Recent neurocognitive research substantiates this interpretation, as (Dajani and Uddin 2015) identify executive flexibility as a distributed capacity emerging from dynamic interactions between the prefrontal cortex, the anterior cingulate cortex, and the insular salience network—all regions that activate during task-switching and symbolic representation. This convergence between behavioral models and brain architecture underscores the central claim that dual-domain fluency emerges not from additive domain expertise, but from the brain's capacity to organize representational frameworks across contexts. The evidence undermines static models of cognitive strength and points to a systems-level configuration in which representational agility (i.e., the ability to move across verbal, numerical, visual, and formal representations while preserving relational meaning) functions as a core mechanism of integration.

If executive function enables the mind to process symbolic structures, then the core question is: what kinds of symbolic systems must it navigate, and how do people learn to translate between them? Although frequently cast as separate domains, verbal and numerical reasoning rely upon shared underlying cognitive processes that foster relational mapping, structural abstraction, and analogical transfer. This overlap finds further support in Spelke and Kinzler's (2007) argument that, although evolutionarily distinct, the cognitive systems responsible for processing numbers, spatial relations, and linguistic forms are cognitively integrated through symbolic convergence. This integration exemplifies a propensity for aligning discrete symbolic systems into a common relational frame. (Nathan and Walkington 2017) affirm this view by demonstrating that students who can transfer structural reasoning from algebraic expressions to sentence logic exhibit deeper understanding in both domains. The authors' findings suggest that the symbolic barrier between mathematics and language dissolves when learners are asked to map underlying schemas rather than surface information. Carroll's (1993) factor-analytic synthesis of cognitive abilities further supports this integration and reveals sharp correlations between verbal analogical reasoning and numerical problem-solving. Viewed collectively, these studies support the idea that dual-domain fluency enables abstraction across symbolic systems, not as a byproduct of education but as a primary cognitive function rooted in relational structure and representational flexibility.

### 2.5 Neuroscience of cognitive integration

The ability to abstract across symbolic systems suggests that the human mind operates on an architecture more integrated than the one implied by the mistaken belief that linguistic and quantitative reasoning are hardwired into separate hemispheres. Although popular accounts continue to portray the left hemisphere as verbal and the right as spatial or numerical, neuroimaging research challenges this divide. In a comprehensive resting-state fMRI study of over one thousand participants, (Nielsen et al. 2013) found no evidence for the left-brain and right-brain dichotomy in functional connectivity. Instead, their findings showed that high-performing people exhibit strong bilateral activation and cross-hemispheric synchronization in the frontal and parietal regions of the brain—the networks involved in symbolic reasoning, task-switching, and abstract rule formation. Also, in mapping patterns of brain asymmetry, (Toga and Thompson 2003) found that even though lateralization takes place at the anatomical level, symbolic cognition relies more on integrative flow between hemispheres, particularly through commissural structures such as the corpus callosum.

Similarly, (Deary et al. 2010) showed that variability in verbal-numerical reasoning is better explained by whole-brain efficiency than by hemispheric specialization. This pattern reveals the neural coordination underlying DDCF. The left inferior frontal gyrus contributes to semantic encoding and symbolic translation, enabling meaning to be reformulated across linguistic and formal structures. The intraparietal sulcus supports quantitative abstraction and magnitude mapping, anchoring numerical reasoning within relational frameworks. The dorsolateral prefrontal cortex facilitates cognitive flexibility and task switching, permitting representational shifts across symbolic formats. The medial prefrontal cortex supports layered reasoning and integrative evaluation, coordinating meaning across domains. Together, these regions form a synchronized network that stabilizes cross-symbolic reasoning during complex problem solving.

Semantic encoding processes associated with the inferior frontal gyrus operate in continuous interaction with quantitative abstraction mechanisms in the intraparietal sulcus. These domain-specific operations are dynamically coordinated by executive control systems in the dorsolateral prefrontal cortex, which enable task switching and representational flexibility across symbolic formats. At a higher level, the medial prefrontal cortex supports integrative evaluation, allowing outputs from these systems to be synthesized into coherent reasoning. The capacity for dual-domain fluency therefore depends not on isolated modules, but on the stability and efficiency of interactions across these regions.

At the behavioral level, the operation of this network manifests as variability in how individuals coordinate symbolic systems. While dual-domain fluency originates in the integrated functioning of distributed neural systems, its expression varies considerably across individuals. These differences do not reflect fixed levels of intelligence but a functional variability in how people encode, restructure, and retrieve abstract representations across domains. Here, Carroll's (1993) three-stratum theory offers a foundational framework, as it sets fluid reasoning, verbal comprehension, and quantitative reasoning as distinct yet correlated abilities that together support symbolic transfer. Moving beyond this structural view, (Rose et al. 2013) challenge standardized models of ability altogether, arguing that cognitive performance is highly context-dependent and shaped by dynamic patterns of cognitive development. The authors' research in dynamic skill theory reveals that integrative thinking is often masked by traditional assessments which separate competencies instead of tracking how they interact in real time. Likewise, (Gardner 2006) has challenged fixed models of intelligence and proposed that multiple intelligences, including logical-mathematical and linguistic, operate in combination to solve complex problems, especially in environments requiring symbolic re-representation. These additional perspectives further position dual-domain fluency as a situated capacity that emerges when people engage symbolic forms through structure, analogy, and context-dependent reasoning.

### 2.6 Instructional implications

The emergence of dual-domain fluency does not depend solely on cognitive architecture or neural integration. It also depends on whether, or how, educational systems support or impede the coordination of symbolic forms. Curricula that separate mathematics from language arts and emphasize procedural repetition over structural reasoning weaken students' ability to recognize abstract parallels across domains. As (Wang and Degol 2017) observe, tracking systems based on early performance indicators typically consolidate into rigid pathways, channeling students into narrowly defined ability groups—which hinders cross-domain synthesis. This institutional sorting, they argue, contributes to enduring disparities in integrative reasoning, particularly among students from underrepresented backgrounds. (Alonzo and Gotwals 2012) extend this criticism by explaining how instructional misalignment across subjects prevents many students from mapping structural patterns between symbolic systems. Even when interdisciplinary content is introduced, it often lacks the conceptual scaffolding needed to support symbolic translation. Heller and Reif's (1984) work in physics education echoes this concern and shows that students trained only in numerical problem-solving fail to apply those insights to verbal analogs—a clear indication that symbolic fluency cannot be presumed to transfer automatically across formats. In educational contexts that reward domain-specific accuracy while sidelining relational abstraction, symbolic coordination is not simply neglected, it is structurally stifled (Alonzo and Gotwals, 2012; Heller and Reif, 1984; see also Wang and Degol, 2017).

Similarly, recent research on creative cognition and brain network development indicates that educational environments encouraging flexible representational use support the development of integrative reasoning capacities in school-age learners (Duval, 2006). Such instructional shortfalls carry measurable cognitive consequences for students. Research on analogical transfer shows that the ability to map relational structures across domains is teachable and predictive of advanced reasoning skills (Richland et al., 2007). Yet most curricula undervalue this capacity by teaching subjects as isolated fields of knowledge rather than as interconnected systems that share symbolic structures. In a longitudinal study, (Schwartz et al. 2012) found that students taught to explain why a math solution works, rather than merely how, were more likely to transfer that understanding to scientific contexts, where structural reasoning is paramount. This kind of relational insight, the authors suggest, does not arise from rote exposure but from clear instruction that draws analogies across representational formats. Along similar lines, (Gentner and Forbus 2011) demonstrated that even young learners can extract structural correspondences between stories and equations when guided to focus on underlying relationships instead of surface details. Indeed, when instruction cultivates this kind of cross-symbolic literacy, students begin to treat mathematical, verbal, and visual systems not as isolated things but as interchangeable tools for thought. This developmental trajectory is summarized in Table 2, a literature-informed conceptual model proposed in this article. The table synthesizes research on representational competence, analogical transfer, and symbolic coordination into a developmental continuum extending from basic decoding to generative symbolic integration. Early stages involve decoding individual symbolic systems, such as interpreting equations or parsing textual arguments. Intermediate phases require bidirectional translation, including reformulating quantitative models into narrative explanations or mapping graphical data onto causal interpretations. Advanced stages involve structural alignment across domains, where learners coordinate symbolic systems to preserve relational meaning. At its most developed level, symbolic integration is generative, enabling individuals to construct models from narratives and articulate narratives through formal representations. The table presents dual-domain fluency as a progressive cognitive synthesis rather than a discrete skill.

**Table 2:** Literature-informed conceptual model of the developmental progression underlying dual-domain cognitive fluency.

Stage	Core process	Cognitive function	Representational scope	Outcome
**Decoding**	Single-system interpretation	Interpreting symbols within a given representational system (e.g., equations, text, graphs)	Isolated representations	Accurate recognition and parsing of symbolic elements
**Representation**	Within-system structuring	Organizing elements into coherent structures within the same system	Structured single-system representations	Formation of internally coherent symbolic structures
**Abstraction**	Cross-system mapping	Translating and aligning representations across different symbolic systems (e.g., equations ↔ text ↔ graphs)	Multiple representations linked	Preservation of relational meaning across formats
**Generative symbolic integration**	Multi-system coordination and construction	Coordinating and integrating multiple symbolic systems to construct new representations	Fully integrated representational systems	Generative reasoning and model construction across domains

This table presents a literature-informed conceptual model proposed in the present article. It synthesizes research on representational competence, analogical transfer, and symbolic coordination into a developmental progression from basic decoding to generative symbolic integration, characterized by increasing coordination across representational systems.

The failure to support symbolic bilingualism weakens students' ability to connect and reframe ideas across different formats, such as equations, diagrams, and verbal explanations. When instruction treats each form as self-contained—as when solving for “*x*” without asking why it matters, or diagramming a sentence without mapping its logic to argumentation—it narrows students' ability to see any structure beyond the surface. What is lost in the process is representational variety but also the cognitive flexibility that emerges from translation itself. Rather than alternating between formats, learners must be guided to make structural comparisons that show what the representations share, and what each clarifies. (Ainsworth 2006) reports that this kind of mapping sharpens students' understanding not only of content, but also of the symbolic systems that shape how knowledge is built. For example, research on multiple representation use shows that students internalize deeper patterns when they shift between formats rather than staying focused in one (Ainsworth, 2006). Yet in many classrooms, such shifts are rare or incidental. (Nathan and Walkington 2017) argue that representational fluency is purely developmental, not innate. It expands through active mapping between story problems, symbolic notation, and visual models.

Otherwise stated, symbolic insight does not emerge from content alone. It is shaped by how students express it. In the absence of such opportunities, students may achieve procedural success without developing transferable understanding. What may be lacking in such cases is what (diSessa and Sherin 2000) have termed “symbol sense”—not the mere ability to recognize symbols but the ability to use them to think. Viewed in this way, what sets cognitive fluency apart is not symbolic knowledge in isolation, but the ability to reframe problems, shift perspectives, and interpret symbolic forms. When this flexibility is neither modeled in instruction nor valued in assessment, schools end up rewarding superficial precision while neglecting the profound cognitive agility that abstraction requires. The result is a type of brittle competence, helpful in ordinary tasks but unhelpful when abstraction is required. According to (diSessa and Sherin 2000), it is in those moments of clear representational instability that dual-domain fluency shows its value.

The development of symbolic agility also shapes how learners come to perceive, value, and align themselves with different domains of knowledge. In this broader sense, dual-domain fluency is both a representational skill and a gateway to disciplinary practices that allow learners to engage in the knowledge practices that define disciplinary thought. When students are encouraged to move fluidly between verbal and mathematical representations, or between narrative and quantitative forms, they come to understand how each operates but also how reasoning within a given domain is structured and practiced. As (Boaler and Dweck 2022) argue, this shift is critical for lifting identity-based barriers in fields like mathematics, where symbolic manipulation is often mistaken for deeper understanding. Students who work with mathematical symbols only in abstract form may never see math as a language for explanation or discovery. Yet when those symbols are grounded in real-world contexts or narratively explained through diagrams and models, students begin to understand and adopt the interpretive frameworks and inferential structures that define disciplinary expertise. (Lee and Sherin 2006) emphasize that representational fluency plays a pivotal role in this process, particularly in science classrooms where translating across models, graphs, and written explanations enables students to deepen inquiry and make disciplinary thinking visible. (Hall and Stevens 1994) extend this insight by showing that students identify with a discipline when they frequently use the representational tools it values.

### 2.7 Equity, access, and symbolic fluency

If dual-domain fluency sharpens thought and reshapes disciplinary associations, it also redraws the boundaries of access. When instruction canonizes a single modality, it narrows the range of acceptable reasoning and filters who gets to count as competent. This filtration is not a neutral sorting of aptitude but rather a distortion of opportunity: students whose strengths lie in alternate forms of expression are often misclassified as low-ability learners, not because they misunderstand the material but because the dominant representational modality used in instruction does not align with how they express or organize knowledge (Moschkovich, 2015). Studies in multilingual classrooms make this visible. When students are allowed to translate across representations—story problems into sketches, graphs into spoken explanations—they recover agency over the symbolic terrain itself (Domínguez, 2011; Moschkovich, 2015). In this symbolic recoding operation, representational fluency functions as a cognitive asset but also as a structural countermeasure to the exclusionary norms of monolithic instruction. As (Gutiérrez 2009) argues, equity in education is not achieved by simply including more voices, but by redefining the very conditions under which participation is meaningful. On this account, dual-domain fluency functions as a means of disrupting inherited boundaries around who is allowed to engage, reason, and be recognized within academic spaces.

This critique becomes clearer when paired with a concrete example that transcends format or modality: the Pythagorean theorem. The symbolic power of dual-domain fluency does not lie in accumulating formats or modalities but in transfiguring meaning across them. And nowhere is this principle more vividly illustrated than in that geometric insight. A single mathematical truth, the Pythagorean theorem can be expressed as an equation, a diagram, or a verbal relational rule. For some learners, its logic unfolds numerically: *a*^2^ + *b*^2^ = *c*^2^. For others, it takes shape visually, through the square areas that define the sides of a right triangle. Still others grasp it in language: the square of the hypotenuse equals the sum of the squares of the legs. But what connotes deep understanding is the ability to toggle among modalities while maintaining relational invariance and recoding the underlying form. Symbolic translation of this kind is not ancillary to cognition; it is fundamental to it. So when schools fail to foster this type of symbolic translation, they short-circuit the very neural architecture that facilitates abstraction, reasoning, and generalization (Bober-Irizar and Banerjee, 2024; Gain and Siegelmann, 2019; Hooshyar et al., 2024; Nathan and Walkington, 2017; Zhao et al., 2024).

### 2.8 Designing instruction for symbolic fluency

The evidence presented suggests that dual-domain fluency is anchored in well-documented neural mechanisms, responsive to structured pedagogical intervention, and predictive of several differential labor market outcomes. A cognitive apparatus capable of translating across symbolic systems—mathematical and linguistic, visual and conceptual—requires more than subtle curricular reform. It requires reframing what educational systems value, how credentials obscure or reflect cognitive ability, and how employers interpret intellectual adaptability. Empirical findings from neuroscience (Dehaene et al., 2021; Immordino-Yang and Damasio, 2007), instructional design (Goldstone and Son, 2005), and cognitive adaptability research (Richland and McDonough, 2010) converge on a core insight. Learning environments that promote symbolic transfer do not merely enhance academic performance. They cultivate the adaptive reasoning aptitudes that enable civic agency and upward mobility. The following section traces the implications of this insight across two areas: the organizing logic of instructional systems and the broader institutional conditions (credentialing practices, hiring protocols, equity policies) that shape access to opportunities.

As the curricular goal shapes what gets evaluated, it also shapes how intelligence is defined. When symbolic fluency is treated as a peripheral enrichment reserved either for the honors track or interdisciplinary electives, it sends a cognitive message that abstraction is optional rather than elemental. But mounting evidence suggests the opposite. Symbolic reasoning, particularly when nurtured across domains, builds the generative schemata that enhances flexible problem-solving and far transfer (Rittle-Johnson and Schneider, 2014; Schwartz et al., 2011). Instructional goals that prioritize representational versatility, or treat it not as a capstone of mastery but as a developmental pathway, extend the schemata that support cross-contextual reasoning, adaptive reframing, and efficient cognitive load management. The implication is that when instructional goals emphasize symbolic versatility and conceptual transfer, the classroom shifts from a staging ground for isolated expertise to a rehearsal space for symbolic exploration. This shift requires a reframing of instructional design. Symbolic translation must no longer be treated as a byproduct of deep understanding but as the primary process through which such insight gradually develops. However, the reframing of symbolic translation as a primary mechanism of learning requires more than adding representations. It requires rethinking how depth is conceived in the first place. Too often, efforts to foster domain-specific competence conflate mastery with isolation, as if students learn mathematics or language arts best when insulated from competing forms of representation. But it is precisely this kind of segregation that hinders cognitive development (Alibali and Nathan, 2012; Nathan et al., 2014; Richland et al., 2007).

On the other hand, exposure to structurally comparable ideas expressed through different modalities, such as equations, graphs, and rhythmic patterns, helps learners to form higher-order abstractions that transcend all disciplinary boundaries (Alibali and Nathan, 2012; Holyoak, 2012). Abstractions formed through representational translation allow students to identify underlying structures in unfamiliar problems. Notwithstanding its many benefits, symbolic abstraction is often regarded as an added representational layer that could burden working memory. Yet research shows that when introduced in a well-sequenced manner, it does not increase cognitive load. On the contrary, tasks that integrate multiple representations allow each representation to scaffold and stabilize the others, thereby reducing cognitive strain (Nathan et al., 2014).

What makes the neglect of symbolic versatility in mainstream classrooms particularly consequential is its disproportionate impact on students who are already structurally marginalized. Curricular models that postpone abstraction until late stages of instruction, or restrict it to honors pathways, treat representational fluency as a reward for previous achievement rather than a precondition for deeper learning (Boaler, 1997). This sequencing error is not pedagogically neutral. It compounds existing inequities by depriving underrepresented students of early access to the tools that enable generalization, transfer, and upward mobility within symbolic systems. The cost is both cognitive and social, as students who are steered toward low-track classrooms that favor procedural routine over conceptual reasoning are less prepared to develop the cognitive flexibility associated with interdisciplinary thinking. Several empirical studies show that such instructional stratification reinforces surface-level processing strategies, discourages analogical reasoning, and limits the formation of transferable schemata (Gaspard et al., 2018; Richland et al., 2007; see also Rittle-Johnson et al., 2009 and Rittle-Johnson et al., 2016). While such effects may seem to reflect a temporary pedagogical delay, they constitute a structural restriction on who gets to engage with symbolic abstraction, and how early.

To position symbolic fluency at the center of instructional design is to reorient pedagogy around the processes that make abstraction usable, transferable, and teachable. Classrooms that embed symbolic translation from the outset cultivate learners who are fluent within domains but also able to shift between them by adapting structures and abstracting principles across contexts. What sets these learners apart is not an innate gift but an early opportunity to develop cross-representational schemata—something that research has shown can be systematically nurtured through analogical scaffolding and multimodal instruction (Alibali and Nathan, 2012; Gaspard et al., 2018; Gentner, 2010; see also Richland and McDonough, 2010). Instructional approaches that ignore the value of symbolic translation default to systems where procedural consistency takes precedence and knowledge transfer is incidental. Embedding symbolic translation into instructional design instead establishes symbolic reasoning as the generative core of learning rather than its accessory.

### 2.9 Structural constraints

Despite its central role, access to symbolic reasoning remains essentially uneven. Educational tracking systems, typically framed as tools for student placement, often function instead as structural constraints that limit access to symbolic reasoning. Far from being neutral instruments of academic differentiation, these systems operate as cognitive gatekeepers that determine who receives instruction that enhances abstract reasoning and who ends up relegated to procedural repetition (Domina et al., 2019; Oakes, 2005). Whenever representational fluency is reserved for upper tracks while lower tracks prioritize procedural compliance, instructional disparity turns into a mechanism for restricting cognitive development. This restriction has real consequences that extend beyond the classroom. Research shows that early placement in low-track programs correlates with long-term underrepresentation in cognitively demanding professions, even when initial academic differences are minimal (Boaler, 1997; Oakes, 2005). In this sense, tracking operates not as a system for differentiating potential, but as an institutional pattern that preselects who is prepared to design systems and who is expected to operate within them (Domina et al., 2019).

The asymmetry embedded in tracking systems is reinforced by curricular content and the types of cognitive tasks students are repeatedly asked to perform. Upper-track classrooms foster inferential reasoning, symbolic manipulation, and open-ended problem framing—skills that align with the demands of highly technical or creative professions. By contrast, lower-track classrooms promote rote procedure, linear execution, and answer conformity, all of which condition students to operate in settings where compliance is preferred to conceptual autonomy (Oakes, 2005). Over time, these asymmetries begin to mirror the epistemic divisions of the workplace itself: between those who are trained to design frameworks and those who are trained to follow them. As this stratification deepens, people come to adopt cognitive roles that replicate and legitimize existing labor divisions (Ansalone, 2010; Oakes, 2005; Rosenbaum, 2004).

Although defenders of tracking claim that it allows students to learn at their own pace, their logic conceals how early placements restrict access to abstract reasoning and treats symbolic reasoning less as a teachable skill than a fixed trait. This dynamic produces a form of instructional predestination: students who find themselves in lower tracks rarely get the conceptual scaffolding necessary to progress to higher tracks. More consequentially, tracking systems impose lasting constraints on academic identity, self-efficacy, and long-term career trajectories (Legette and Kurtz-Costes, 2021).

### 2.10 Credentialing systems

Academic credentials are seen as static indicators of achievement, but they are designed artifacts that reflect institutional decisions about knowledge and validation methods. This design has traditionally compartmentalized cognitive achievement into categories such as STEM or the humanities, verbal or quantitative, and overlooked integrative reasoning as a distinct, assessable aptitude. Dual-domain cognitive fluency (DDCF) highlights this misalignment and emphasizes the need for credentialing systems that recognize interdisciplinary competencies. Recent studies underscore the potential of design thinking as a way of developing interdisciplinary curriculum (Wang, 2024). Concurrently, the rise of micro-credentials reflects a shift toward recognizing competencies that transcend traditional disciplinary boundaries (Ahsan et al., 2023). (Ahsan et al. 2023) argue that micro-credentials offer flexible, targeted learning opportunities that complement traditional degrees and allow for the validation of skills aligned with DDCF. For example, in its policy brief on credentials innovation, the Organization for Economic Co-operation and Development (OECD) emphasizes how micro-credentials could offer broader interdisciplinary curricula or additional skills to students in a way that addresses the evolving demands of the labor market (OECD, 2021). And according to a report published in 2012, without such reforms, schools risk perpetuating narrow competence frameworks that overlook the cognitive flexibility most predictive of innovation and lifelong success (National Research Council, 2012).

But even as new credentialing models gain traction, the assessment infrastructure needed to validate such integrative proficiency remains underdeveloped. Conventional evaluations tend to prioritize content recall, procedural accuracy, or domain-specific output—metrics that obscure the cognitive transitions students must make when navigating across symbolic systems. DDCF entails fluidity between modalities: the capacity to shift from mathematical abstraction to verbal interpretation, or to translate conceptual insight into quantitative models. Assessing such fluency requires instruments that capture not what students know in isolation, but how they coordinate representations, identify structural parallels, and reframe problems in interdisciplinary terms. As (Wang 2024) argues, curriculum innovations grounded in design thinking must be matched with evaluative methods that reward synthesis. The problem is that such assessment reforms are most often constrained by current institutional norms, such as credit hour systems, standardized testing protocols, and accreditation requirements that privilege coverage over conceptual transfer. While micro-credentials are often positioned as a viable alternative, the OECD's (2021) report indicates that the true potential of micro-credentials is in pairing modular content with assessment designs that capture integrative and transferable reasoning across disciplinary and symbolic boundaries.

## 3 Study design considerations

The present section outlines a program of empirical work designed to test dual-domain cognitive fluency as a measurable and trainable construct. Rather than enumerating abstract possibilities, the aim is to specify a set of concrete study architectures that make the construct observable in classroom and laboratory contexts. This approach situates the framework within existing traditions of educational research while defining design features that allow symbolic integration to be distinguished from domain-specific proficiency. Educational practice has long experimented with forms of instruction that link, rather than isolate, symbolic systems. Integrated curriculum models, Montessori environments, and interdisciplinary programs such as those associated with the International Baccalaureate have each attempted to coordinate verbal, quantitative, and visual representations within coherent learning experiences. Empirical syntheses suggest that such approaches can support academic and nonacademic development, although effects vary with implementation quality and assessment alignment. These traditions do not isolate a construct equivalent to dual-domain cognitive fluency, yet they provide pedagogical precedents for instructional environments in which representational coordination is treated as a central feature of learning rather than a peripheral skill.

Educational practice has long explored instructional models that deliberately integrate, rather than separate, symbolic systems. Montessori education, for example, emphasizes coordinated development of verbal, quantitative, and sensory representations through structured, hands-on learning environments that support abstraction through manipulation (Lillard, 2017). Interdisciplinary frameworks such as the International Baccalaureate explicitly position cross-domain integration as a curricular objective, requiring students to connect mathematical, linguistic, and conceptual knowledge across subject boundaries (Drake and Reid, 2018). More broadly, research on integrated curriculum indicates that learning environments organized around conceptual coherence rather than disciplinary segmentation can support deeper understanding and transfer, although effects depend on implementation quality and alignment with assessment practices (Beane, 1997; Drake and Burns, 2004). These traditions do not isolate dual-domain cognitive fluency as a formal construct, yet they provide established pedagogical contexts in which systematic coordination across symbolic systems is already treated as a central feature of learning.

### 3.1 Designing instruction for symbolic fluency

A first empirical entry point lies in structured translation tasks that require learners to move between symbolic systems while preserving relational meaning. Bidirectional translation paradigms provide a direct behavioral foundation for this approach. Participants can be asked to reformulate mathematical word problems into algebraic expressions and then reconstruct those expressions into coherent verbal explanations. Performance can be evaluated for preservation of relational structure, semantic compression, and symbolic alignment, allowing researchers to observe how meaning is maintained or distorted during representational recoding (De Smedt et al., 2011; Koedinger and Nathan, 2004; Ainsworth, 2006). To make these processes observable in instructional settings, a classroom-based design can embed such translation cycles within a structured unit. For example, middle-school students may complete a 6-week module on proportional reasoning in which each lesson requires expression of quantitative relationships across algebraic notation, diagrammatic representation, and written explanation. Instructional prompts would explicitly direct attention to structural equivalence across representations, rather than surface features of any single format. This approach builds on established findings that learning with multiple representations depends critically on learners' ability to align relational correspondences across formats rather than encounter them in parallel (Ainsworth, 2006; Rittle-Johnson et al., 2009; Schwartz et al., 2012).

Complementary paradigms may include analogical mapping tasks that require participants to align structural relations across symbolic systems. Legal arguments can be paired with formal proofs, narrative causality can be mapped onto conditional logic, and argument diagrams can be translated into quantitative flow structures. Such tasks extend beyond domain-specific performance to examine whether learners preserve conceptual invariance across representational formats (Gentner and Smith, 2012; Richland et al., 2007; Richland and McDonough, 2010). Performance within these paradigms can be evaluated using a multi-component scoring framework. Accuracy measures capture correctness within individual representations, while structural alignment indices assess whether equivalent relationships are preserved across formats. Latency measures track the efficiency of recoding, and explanatory coherence scores evaluate the clarity and completeness of verbal reconstructions. Together, these indices make symbolic integration behaviorally traceable and allow researchers to distinguish integrative fluency from domain-specific proficiency. Over repeated administrations, such measures can reveal developmental growth, learning sensitivity, and the stability of symbolic coordination as a cognitive capacity (Koedinger et al., 2013; Schneider et al., 2011).

### 3.2 Study design features

Empirical research on dual-domain cognitive fluency requires designs capable of capturing both developmental emergence and structural stability across contexts. A mixed-methods architecture that integrates cross-sectional comparison with longitudinal tracking offers the most comprehensive foundation. Cross-sectional designs can examine variation across age groups, educational pathways, or disciplinary specializations, offering initial insight into when symbolic fluency consolidates and how it differentiates from isolated verbal or quantitative skill. Such comparisons establish descriptive baselines and reveal distributional patterns across learner populations. They also allow examination of differences across instructional environments, including those that emphasize integrated or interdisciplinary learning relative to more domain-segmented curricula, thereby situating symbolic coordination within existing educational practices.

Longitudinal designs extend this work by tracing whether early symbolic flexibility predicts later reasoning proficiency and interdisciplinary transfer (Rittle-Johnson et al., 2009; Schneider et al., 2011). Students may be followed across multiple academic years, completing translation and analogical mapping tasks at structured intervals while participating in reflective interviews that document metacognitive strategy formation. These layered data sources allow researchers to examine how symbolic coordination evolves through instruction, practice, and cognitive maturation. Hybrid designs of this kind align with developmental models of transfer and structural abstraction (Schwartz et al., 2012). They also permit triangulation across behavioral performance, narrative explanation, and academic outcomes, enabling researchers to determine whether dual-domain fluency functions as a coherent developmental construct that supports learning generalization across symbolic environments.

### 3.3 Construct validity and psychometric strategy

Establishing dual-domain cognitive fluency as a distinct construct requires psychometric modeling capable of differentiating integrative reasoning from traditional domain abilities. Confirmatory factor analytic approaches provide a rigorous framework for testing whether symbolic translation, cognitive flexibility, and layered reasoning load onto a unified latent structure independent of verbal or quantitative proficiency (Kline, 2015; Kovacs and Conway, 2016; McGill and Dombrowski, 2019). Item pools can be constructed from translation tasks, analogical mapping protocols, and argument deconstruction exercises, each scored for structural preservation across representational shifts. Such scoring moves beyond surface accuracy to capture relational coherence during symbolic transformation. Parallel assessment batteries may include verbal comprehension, mathematical fluency, and executive function measures, allowing researchers to evaluate convergent and discriminant validity. Through this modeling architecture, dual-domain fluency can be positioned as an integrative cognitive capacity with measurable boundaries. Evidence for construct validity would be strengthened if the proposed latent factor accounts for variance in cross-representational performance beyond what is explained by domain-specific abilities, consistent with prior work on general cognitive integration and executive coordination (Kovacs and Conway, 2016). Neurocognitive evidence can further strengthen construct validation. Functional imaging paradigms and electrophysiological measures can examine whether symbolic translation tasks recruit neural systems associated with executive coordination and representational integration, including regions implicated in semantic encoding and cognitive control (Dajani and Uddin, 2015; Fedorenko et al., 2013). Converging behavioral and neural evidence of this kind provides the evidentiary scaffolding required to position dual-domain fluency as a measurable dimension of cognition grounded in performance structure and brain function.

### 3.4 Instructional intervention trials

If dual-domain cognitive fluency is to extend beyond laboratory measurement, its trainability must be examined through structured instructional intervention. Curriculum-based trials offer a direct mechanism for testing whether symbolic integration can be cultivated through pedagogical design. Experimental cohorts can engage in translation-rich learning environments where algebraic solutions are rewritten as narrative explanations, argumentative structures are diagrammed visually, and statistical findings are reframed as policy interpretations (Alibali and Nathan, 2012; Gentner, 2010; Richland and McDonough, 2010). Control cohorts may receive conventional domain-bounded instruction aligned with standard curricular sequencing. A concrete implementation can be structured as a 6-week quasi-experimental unit in which treatment students complete repeated translation cycles across symbolic formats, with explicit prompts directing attention to relational alignment. Comparison students would engage with the same content, duration, and practice volume but without structured cross-representational translation. This design isolates the contribution of symbolic coordination from general instructional exposure and aligns with prior research demonstrating that instructional guidance is necessary for learners to benefit from multiple representations (Ainsworth, 2006; Rittle-Johnson et al., 2009).

Comparative analysis across these conditions can evaluate whether translation training strengthens transfer performance, abstraction capacity, and representational flexibility. Assessment architectures may include proximal achievement tasks, delayed transfer evaluations, and metacognitive reflection protocols that document how learners reorganize knowledge while navigating symbolic systems. Additional indicators, such as cognitive load perception and learner confidence, can provide insight into how integrative reasoning develops experientially (Schneider et al., 2011; Sweller et al., 2019). Intervention studies of this kind allow researchers to determine whether dual-domain fluency emerges solely through maturation or whether it expands through deliberate instructional design. In doing so, such designs connect theoretical architecture to pedagogical application and position symbolic integration as a teachable dimension of cognitive development.

## 4 Discussion

The false dichotomy between verbal and quantitative minds has long shaped educational systems and popular beliefs about intelligence. This framing encourages specialization at the expense of cognitive versatility and undervalues skills that bridge symbolic systems. The dual-domain cognitive fluency framework offers a compelling, data-supported alternative model that defines intelligence as the capacity to integrate meaning across numerical, verbal, and visual modalities. This integrative capacity occupies a central position within complex reasoning and professional problem solving. This form of reasoning promotes abstraction, knowledge transfer, and conceptual synthesis. In fields ranging from data science to public policy, such abilities are essential for generating cross-cutting insight. As research shows, people who engage in versatile thinking are more likely to generate creative solutions and adapt to evolving careers (Fischer and Bidell, 2007).

This article defines dual-domain cognitive fluency as a new framework while situating it within decades of foundational research spanning cognitive science, educational psychology, and neuroscience. A central strand of this research is dual-process theory, which holds that effective reasoning emerges from the interplay of intuitive and analytical systems that can alternate between pattern recognition and deliberate analysis (Stanovich and West, 2000). This interplay fosters the ability to shift across visual, quantitative, and verbal modalities. Neuroscientific findings further support this connection and link integrative reasoning to increased activity in brain areas associated with abstraction, symbolic manipulation, and executive control (Davis et al., 2008; Ralph et al., 2017). Within the DDCF framework, such coordinated activation reflects the neural orchestration required to translate, align, and stabilize meaning across symbolic systems during complex reasoning.

Educational systems remain out of step with the cognitive skills that dual-domain fluency involves and the research that supports its value. Too many schools continue to prioritize domain-specific recall over integrative reasoning and to award credentials that reflect content exposure rather than conceptual synthesis. As a result, learners who excel in cross-domain reasoning often go unrecognized or fall through evaluative gaps that reward compartmentalized knowledge over cognitive flexibility. The same pattern holds in professional contexts, where symbolic integration is often overlooked, particularly when analytical reasoning is paired with narrative interpretation (Wang and Degol, 2017).

Addressing these problems requires changes to instruction and assessment. Institutions must reimagine instruction by embedding symbolic translation into curricula and developing assessments that value abstraction, synthesis, and structural insight. Since dual-domain cognitive fluency reflects the structure of intelligent reasoning, ignoring it sustains a narrow view of cognition. Future research should examine how dual-domain fluency develops across age groups and instructional contexts. Initial empirical efforts may begin with translation-based performance batteries, symbolic recoding latency measures, and structural alignment scoring protocols designed to capture integrative reasoning in action. Longitudinal studies might trace the emergence of symbolic flexibility over time, while controlled interventions could evaluate the effects of multimodal instruction on symbolic translation, cognitive flexibility, and integrated reasoning (Koedinger and Nathan, 2004; Richland and McDonough, 2010). Coordinated multi-site investigations spanning educational psychology, cognitive science, and neuroscience would further accelerate construct validation and encourage collaborative model refinement. Another line of inquiry could investigate how DDCF influences student motivation, particularly in environments where traditional metrics undervalue representational agility. The core argument advanced in this paper centers on the necessity of studying dual-domain fluency as a measurable dimension of intelligence that remains systematically underrepresented in research and instruction. Its cognitive architecture links abstraction, translation, and adaptability in ways that elude standard assessments and fragmented curricula. If future research confirms that symbolic agility supports learning transfer and disciplinary reasoning, then dual-domain cognitive fluency may reshape how educational psychologists conceptualize higher-order thinking.

## Data Availability

The original contributions presented in the study are included in the article/supplementary material, further inquiries can be directed to the corresponding author.
